# Nanoparticles Enhance In Vitro Micropropagation and Secondary Metabolite Accumulation in *Origanum petraeum*

**DOI:** 10.3390/nano15191496

**Published:** 2025-09-30

**Authors:** Tamara S. Al Qudah, Rida A. Shibli, Rund Abu-Zurayk, Mohammad Hudaib

**Affiliations:** 1 Hamdi Mango Center for Scientific Research, The University of Jordan, Amman P.O. Box 11942, Jordan; t.alqudah@ju.edu.jo (T.S.A.Q.); r.shibli@ju.edu.jo (R.A.S.); 2 Department of Horticulture and Crop Sciences, Faculty of Agriculture, University of Jordan, Amman P.O. Box 11942, Jordan; 3 Faculty of Pharmacy, The University of Jordan, Amman P.O. Box 11942, Jordan; m.hudaib@ju.edu.jo

**Keywords:** *Origanum petraeum*, microshoots, secondary metabolites, essential oils, silver nanoparticles, copper nanoparticles, GC-MS analysis

## Abstract

*Origanum petraeum* Danin, an endemic medicinal shrub from Jordan, belongs to the Lamiaceae family and possesses significant pharmaceutical potential, yet its secondary metabolite profile remains largely unexplored. This study evaluated the effects of two types of nanoparticles, silver (Ag) and copper (Cu), on in vitro propagation and secondary metabolite composition in *O. petraeum* microshoots. Sterilized buds were used to initiate in vitro cultures on Murashige and Skoog (MS) medium supplemented with gibberellic acid (GA_3_) at 0.5 mg/L. Microshoots were treated with nanoparticles at concentrations of 0, 25, 50, 100, and 150 mg/L. AgNPs at 100 mg/L promoted growth, increasing the number of microshoots to 11.6 and shoot height to 9.22 cm. Transmission electron microscopy confirmed nanoparticle uptake and translocation, with AgNPs observed in root cells as small particles (≤24.63 nm), while CuNPs formed aggregates in leaves (47.71 nm). GC-MS analysis revealed that nanoparticles altered the volatile composition; 50 mg/L CuNPs enhanced monoterpenes, including α-terpinyl acetate (29.23%) and geranyl acetate (12.76%), whereas 50 mg/L AgNPs increased sesquiterpenes, such as caryophyllene oxide (28.45%). Control in vitro cultures without nanoparticles showed simpler profiles dominated by caryophyllene oxide, while wild plants contained both monoterpenes and sesquiterpenes, with eudesm-7(11)-en-4-ol (25.10%) as the major compound. Nutrient analysis indicated that nanoparticles influenced nutrient composition in microshoots. This study is the first to report nanoparticle-assisted growth and essential oil composition in *O. petraeum*, demonstrating their potential to enhance growth and secondary metabolite production for pharmacological and biotechnological applications.

## 1. Introduction

Medicinal plants are herbs that contain various active therapeutic compounds; however, they currently face numerous threats in their habitats across different geographical regions [1]. Jordan possesses a rich diversity of medicinal plants and genetic resources, which are unique in their medicinal components; yet, many of these valuable species are now at risk of extinction in their natural environments [2,3]. The *Origanum* genus is known for its wide range of antioxidant, antibacterial, antifungal, antiparasitic, and antithrombin properties, primarily found in the Mediterranean region [4,5].

*Origanum petraeum* Danin is a threatened wild shrub endemic to limited areas of Petra, Jordan, making it a unique species within Jordan’s plant genetic resources [6]. It is valued as a medicinal, aromatic, and ornamental plant, particularly by the Bedouins of Petra. In vitro culture techniques provide a viable method for propagating plants that are difficult to propagate traditionally, while also serving as a tool for studying secondary metabolites [7,8,9]. Recent studies on plant tissue culture have shown the capability of nanoparticles in enhancing propagation and secondary metabolite production [10,11,12].

Metallic nanoparticles, such as Ag, Cu, Fe, and titanium, are employed in biological studies due to their active properties [13]. The exceptional properties of nanoparticles are attributed to their small size, typically ranging from 1 to 100 nm [14]. Nanoparticles have demonstrated multiple functions in the tissue culture of plants, such as enhancing the activity of anticancer and antimicrobial agents, promoting development, and increasing natural products in tissue-cultured medicinal plants [10,14,15]. Nanoparticles (NPs) have also been documented to assist plants in coping with stress, which can further enhance the production of secondary metabolites under adverse conditions [16]. Recently, the application of nanomaterials to increase the elicitation of natural products in plants has received significant attention [15]. Notably, an increase in secondary metabolite production has been observed when using silver nanoparticles (AgNPs) in in vitro plant cultures [17]. Additionally, copper nanoparticles (CuNPs) have been used to enhance natural products and their components, such as phenols, alkaloids, and flavonoids, in *Bacopa monnieri* L. [18], as well as to increase the accumulation of medicinal components in *Hypericum perforatum* L. in vitro cultures [19]. Furthermore, several studies have highlighted the benefits of incorporating nanoparticles into the medium of tissue-cultured plants to support growth and development [17,20].

Overall, the *Origanum* genus has been evaluated in prior research regarding propagation and conservation [21,22]. Some studies have investigated the impact of nanoparticles on growth, such as examining the effect of CuNPs on the growth and physiological characteristics of *Origanum vulgare* [23]. Other studies have utilized extracts from the *Origanum* genus for eco-friendly nanoparticle production [24,25]. However, there is a clear absence of studies addressing the micropropagation and secondary metabolite production of *O. petraeum*, particularly in relation to nanoparticle applications. Thus, the present research was designed to evaluate how silver and copper nanoparticles influence micropropagation and promote secondary metabolite formation in *O. petraeum*.

## 2. Materials and Methods

### 2.1. Plant Source and In Vitro Establishment

The plant material was originally established under a foreign-funded project by the Ministry of Higher Education in Jordan (Project No. AGR/1/13/2019), where in vitro shoots of *O. petraeum* were initiated from axillary buds collected from a wild shrub at the Royal Botanic Garden (RBG), Jordan. No voucher specimen of the plant from which the explants were taken is reported. The original plant occurs in the Dana Biosphere Reserve, Tafilah, Jordan (30°41′15″ N, 35°34′21″ E) [2], and the Royal Botanic Garden holds the authority to provide plant material. The in vitro shoots were subsequently established and subcultured on MS medium [26] supplemented with GA_3_ (0.5 mg/L) and sucrose (30 g/L) until sufficient material was obtained for further experiments. For more detailed information on establishing in vitro cultures from axillary buds, see the previous study [8].

### 2.2. Nanoparticles Applications

Silver nanoparticles (AgNPs, 50–60 nm, 99.9% purity; Product #0121XH, Lot #0121-010424) and copper nanoparticles (CuNPs, 40–60 nm, 99.9% purity; Product #0820XH, Lot #0820-100422) were purchased from SkySpring Nanomaterials, Inc. (Houston, TX, USA) for experimental use. Pre-manufactured nanoparticles were used instead of chemically synthesized nanoparticles to minimize size variability and ensure consistency. Silver (Ag) and copper (Cu) nanoparticles (NPs) were applied to MS media containing in vitro shoots. Each NP concentration (0.0, 25, 50, 100, and 150 mg/L) was incorporated into the MS medium before adding agar. After agar addition, the medium was boiled with continuous stirring and then autoclaved. Once autoclaved, 0.5 mg/L GA_3_ was added using filter sterilization (0.45 µm), followed by further stirring to ensure even NP distribution. The medium was then dispensed into flasks at 100 mL each. Microshoots of previously established in vitro *Origanum petraeum* shoots (1.0 cm each) were subsequently transferred to MS medium containing either AgNPs or CuNPs, and tissue cultured plants were maintained in growth room conditions with a 16 h light/8 h dark photoperiod, at an illumination intensity of 40–45 μmol m^−2^ s^−1^ and 24 ± 1 °C. The growth room was designed and constructed by Al-Maani Company, Amman, Jordan. For each treatment, five independent replicates were used, with each replicate containing three microshoots. Data on shoot height, shoot number, root number, and average leaf number were collected after four weeks of culture.

### 2.3. Transmission Electron Microscopy (TEM) Imaging of Roots and Leaves for Nanoparticle Tracking

Root and leaf samples were obtained from microshoots after one month of cultivation and grown on the previously mentioned MS media containing 50 mg/L of either Cu or Ag nanoparticles (NPs). The 50 mg/L nanoparticle concentration was selected for its balanced effect, avoiding toxicity and ensuring reliable results. This concentration also allows easy comparison across treatments, considering the high cost of TEM analysis. The samples were preserved in 3% glutaraldehyde for 24 h, followed by treatment with osmium tetroxide for one hour to enhance structural contrast. Subsequently, the samples underwent sequential dehydration using ethanol: 70% for 10 min, 95% for 10 min, and 100% ethanol for two cycles of 30 min each. After dehydration, the samples were cleared via propylene oxide treatment for 30 min, and infiltration was subsequently achieved using a 1:1 mixture of resin and propylene oxide for a minimum of three hours or left overnight, followed by pure resin infiltration overnight. The embedded samples were then sectioned, and ultrathin sections of approximately 70 nm were obtained, treated with uranyl acetate and lead citrate, and examined under a transmission electron microscope (TEM) to assess the localization of NPs. For analysis, a Morgagni FEI 268 transmission electron microscope, manufactured in Tilburg, The Netherlands, was used.

### 2.4. Phytochemical Analysis

#### 2.4.1. Essential Oil Extraction Using Hydrodistillation

Air-dried plant materials were obtained from in vitro-derived microshoots of *Origanum petraeum* grown on the establishment MS media mentioned before and having sucrose at 30 g/L and GA_3_ at 0.5 mg/L, and treated with either Cu NPs or Ag NPs using 50 mg/L level for each. Two control groups were included: Control 1 consisted of tissue cultured plants on the same MS medium without nanoparticles, while Control 2 comprised aerial parts of wild *O. petraeum* plants. All samples were dried at 24 °C, and approximately 50 g from different samples underwent distillation via Clevenger-type apparatus. The extracted essential oils were recovered using n-hexane. The oils were put in dark glass vials at 4 °C until further analysis. For GC-MS profiling, each essential oil sample was diluted by mixing 5 μL of oil with 1 mL of GC-grade n-hexane, and the solutions were then injected into the GC-MS system for phytochemical characterization.

#### 2.4.2. Analysis of Essential Oils Using Gas Chromatography with Flame Ionization Detection (GC-FID)

Essential oils were analyzed using a Focus GC system (Thermo Electron, Waltham, MA, USA) equipped with a split/splitless injector (1:50) and a flame ionization detector (FID). Separation was performed on a DB-5 column (30 m × 0.25 mm × 0.25 μm) using a linear temperature program from 60 °C to 250 °C at 3 °C/min, held at 250 °C for 5 min. Injector and detector temperatures were set at 250 °C and 300 °C, respectively. Hydrodistilled oils were directly injected, and relative peak areas were expressed as percentages of the total oil. Each sample was analyzed in triplicate to ensure accuracy.

#### 2.4.3. Analysis of Essential Oils Using GC-MS

GC-MS analysis of essential oils was performed using a Varian Chrompack CP-3800 GC/MS/MS-200 system (Varian, Palo Alto, CA, USA) equipped with a split/splitless injector (1:10) and a DB-5 column (30 m × 0.25 mm × 0.25 µm). Injector, detector, and transfer line temperatures were set at 250 °C, 160 °C, and 230 °C, respectively. The oven temperature was programmed to separate the oil components. The mass spectrometer scanned 35–400 *m*/*z* using electron impact at 70 eV. A series of n-alkanes (C_8_–C_20_) was used to calculate linear retention indices (LRIs) [27], and compound identification was based on MS libraries (WILEY, NIST, ADAMS-2007) and literature LRIs [28]. Major compounds were verified by co-chromatography with authentic standards.

### 2.5. Effect of Nanoparticles on Nutrients Uptake in Microshoots

Samples of the tissue cultured *O. petraeum* microshoots after one month of growth on MS media with no nanoparticles (0.0 NPs, used as the control), or having 50 mg/L CuNPs, or 50 mg/L AgNPs. These samples were analyzed to show NPs influence on the following nutrient (P, K, and Ca) composition in the tissue cultured plants.

For each treatment (control, 50 mg/L AgNPs, and 50 mg/L CuNPs), 0.5 g of dried plant material was digested using a HNO_3_–HClO_4_ mixture following standard protocols [29,30]. After heating until clear, samples were cooled, treated with HCl, and diluted to 25 mL with deionized water. The resulting solutions were analyzed for nutrient content using ICP-OES at 40.68 MHz, with RF power of 1100 W, nebulizer flow of 0.5 L/min, auxiliary gas at 1.7 L/min, plasma gas at 11 L/min, and PMT voltage of 600 V. Mineral concentrations were quantified using calibration curves generated from ICP-OES standards.

### 2.6. Experiments Designations and Analysis of Data

The experiments were arranged in a completely randomized design (CRD), with five replications for the Section 2.2 experiments and three replications for Section 2.5. For Section 2.3 and Section 2.4, analyses were conducted according to the specific experimental procedures described above. Data were analyzed using SPSS software (PSWAD Statistics 18), and mean comparisons were performed at a 0.05 significance level using the Tukey HSD test. The standard error of each mean was also recorded to evaluate variability across treatments.

## 3. Results and Discussion

### 3.1. Effect of the NPs Application on O. petraeum Microshoots Growth

**Figure 1:**
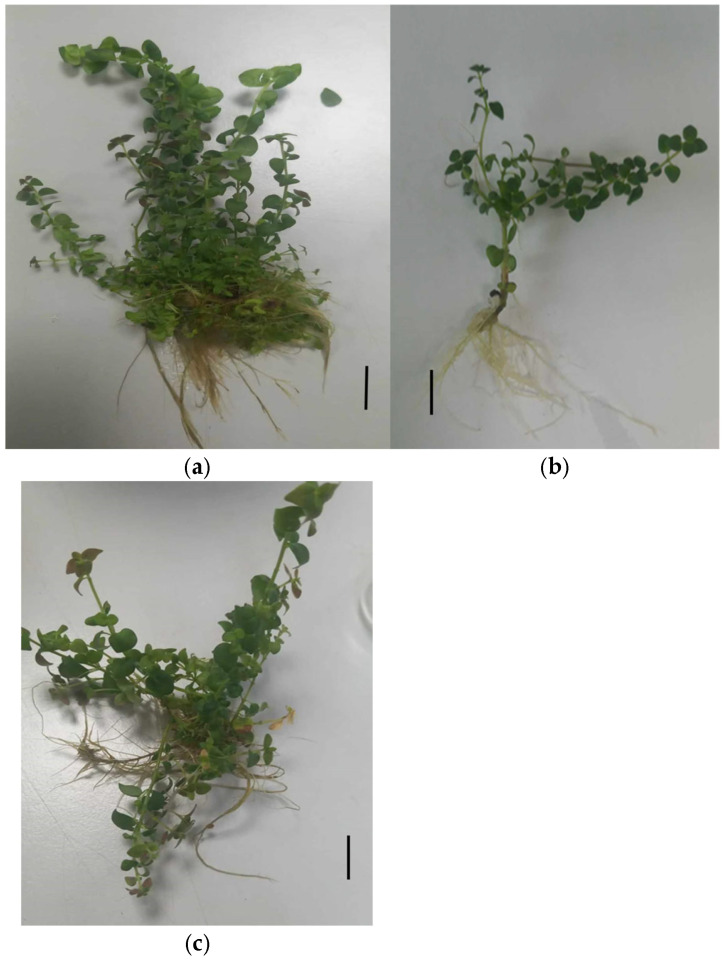
Influence of nanoparticles on growth characteristics of in vitro *O. petraeum* microshoots after 1 month of incubation on MS medium with GA_3_ at 0.5 mg/L and sucrose at 30 g/L. Treatments include (**a**) 100 mg/L AgNPs, (**b**) 0.0 mg/L NPs (control) and (**c**) 50 mg/L CuNPs. Scale bar = 1.0 cm.

As shown in Table 1, AgNPs were applied at different concentrations in the medium to observe their effect on microshoot growth parameters. Interestingly, increasing the concentration of AgNPs up to 100 mg/L effectively boosted the microshoot number, achieving a four-fold increase (11.6 microshoots) and raising shoot height to 9.22 cm as shown in (Figure 1a) compared to the control (0.0 mg/L AgNPs), which produced 2.8 microshoots and a height of 5.2 cm (Figure 1b). This growth improvement was also observed at AgNP concentrations of 25 and 50 mg/L. However, increasing the level to 150 mg/L reduced the microshoot number to 7.6. Root number also increased at 100 mg/L AgNPs, while callus diameter at the base of the microshoots reached 1.8 cm. The highest callus formation occurred at the base of microshoots treated with the highest AgNP concentration, 150 mg/L, resulting in a callus diameter of 2.38 cm. This data suggests the enhancement effect of 100 mg/L AgNPs in the medium. In date palm (*Phoenix dactylifera*), Hayani species, AgNPs stimulated the number of shoots (up to 10.74) and roots (8.4), enhancing growth when applied to MS media at 3.0 mL/L AgNP in combination with 2.0 mg/L BA, 0.1 mg/L NAA, and 0.5 mg/L GA3 [31]. Furthermore, AgNPs at 4.0 mg/L improved in vitro propagation in *Gaillardia pulchella*, resulting in 16 shoots per explant [32].

The positive impact of AgNPs could be attributed to their mode of action, which enhances nutrient uptake and creates a healthier environment by eliminating active hydrogen peroxide (H_2_O_2_), leading to optimal growth parameters [33]. However, in some cases, AgNPs have a negative nature on plant development. For example, Tymoszuk and Kulus (2022) [34] recorded a suppressive effect of AgNPs at different levels when added to *Chrysanthemum* in vitro shoots. This may be due to toxicity at high concentrations, interference with nutrient uptake, or variation in sensitivity among species.

As shown in Table 2, Cu nanoparticles (CuNPs) were added to the media at varying concentrations. The microshoots responded positively to slight increases in CuNP concentration. At 25 mg/L and 50 mg/L, CuNPs gave in an average of 4.8 and 6.8 microshoots, respectively, in comparing with the control treatment (0 mg/L). Shoot height and root numbers showed a similar trend. The tallest shoots were obtained at 50 mg/L of CuNPs, reaching a height of 7.0 cm, with an average root number of 13.8 (Figure 1c). Conversely, higher concentrations of CuNPs negatively impacted plant growth, with plants turning brown at elevated Cu levels, as indicated in Table 2. For instance, a concentration of 150 mg/L produced only 1.6 microshoots, fewer roots, and increased callus diameter at the microshoot base, reaching 2.16 cm. The application of CuNPs in crystalline form at 5 µM enhanced shoot regeneration in *Ocimum basilicum* L. plants, producing 18.7 plantlets compared to CuSO_4_·5H_2_O at 5 µM, which yielded 7.4 shoots [35]. Furthermore, CuNPs improved the in vitro growth of *Capsicum annuum* L. by decreasing reactive oxygen species (ROS) and activation of antioxidant work [36].

Overall, Cu NPs and Ag NPs had distinct effects on the in vitro growth of *O. petraeum* microshoots, likely due to their different modes of action. Cu NPs can lead to increased copper accumulation within the plant, potentially causing toxicity in microshoots, especially at higher concentrations [37]. The use of CuSO_4_ at high concentrations has been reported to exert toxic effects on the regeneration of *Mucuna pruriens* L. [38]. However, such toxicity occurs primarily at elevated concentrations, as observed in *Stevia rebaudiana*, where significant toxicity was recorded with CuO NPs at a rate of 2000 mg/L [39]. The effects of CuNPs and AgNPs on the in vitro growth of *O. petraeum* microshoots highlight the complexity of nanoparticle interactions with plant systems. These findings emphasize the need to optimize nanoparticle concentrations to minimize toxicity while harnessing their potential benefits for plant growth and development. Additional research should be performed to specify the mechanisms of these responses and to determine safe and effective application levels of NPs that should be used with the tissue cultured plants.

### 3.2. Transmission Electron Microscopy (TEM) Imaging of Roots and Leaves for Nanoparticle Tracking in O. petraeum Plant Samples

**Figure 2:**
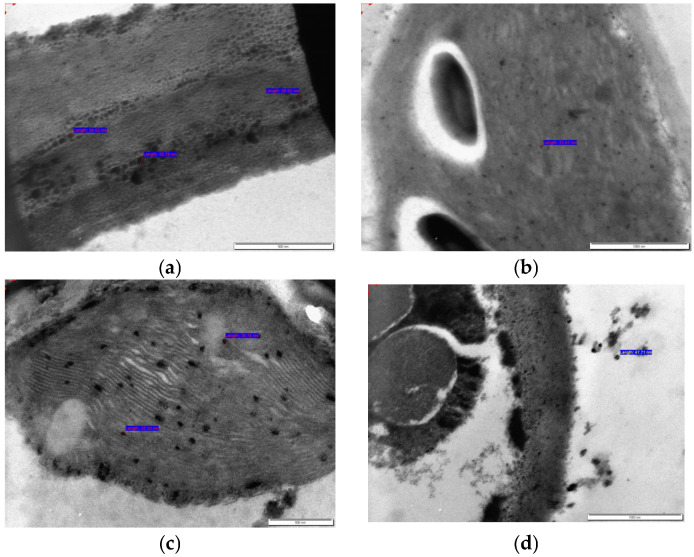
TEM imaging showing the distribution of nanoparticles in *O. petraeum* tissues. (**a**) AgNPs localized within root cortical cells (particle sizes: 28.53, 32.78, and 24.63 nm). (**b**) AgNPs in mesophyll cells of stained leaf tissue (size: 22.62 nm). (**c**) CuNPs localized within root cortical cells (particle sizes: 32.33 and 38.16 nm). (**d**) CuNPs in mesophyll cells of stained leaf tissue (size: 47.71 nm).

Transmission electron microscopy (TEM) images were obtained from microshoot samples treated with 50 mg/L of either AgNPs or CuNPs, focusing specifically on both root and leaf tissues. Each root and leaf sample was processed and imaged individually. As shown in Figure 2a, AgNPs were translocated within root cells, with particle sizes of 28.53, 32.78, and 24.63 nm. The smaller size of AgNPs in the plant samples, compared to those added to the MS medium, is attributed to the plant’s metabolic activity. Biomolecules such as phenolics, flavonoids, and proteins can reduce and stabilize the nanoparticles, preventing aggregation and limiting growth, resulting in smaller and more uniform particles [40,41]. Figure 2b shows AgNPs in stained leaf tissue, measuring 22.62 nm. CuNPs were observed inside root cells (Figure 2c) with a particle size of 32.33 nm, while translocated CuNPs in leaf tissue measured 47.71 nm (Figure 2d). The TEM images were captured after staining the tissues with uranyl acetate, which facilitated the visualization of nanoparticles.

The presence of nanoparticles in the roots and leaves following exposure indicates that they were absorbed by the plant and translocated internally. Nanoparticles are likely taken up by roots—the primary interface between the plant and its medium—through passive diffusion, active transport, or endocytosis [42]. Once inside, they are translocated via the vascular system (xylem or phloem) to other tissues, such as leaves [43]. This movement is affected by factors including the size of the nanoparticles, charge, surface coating, and the physiological state of the plant [44]. Earlier research has demonstrated that AgNPs smaller than 40 nm can pass through root cell walls [45]. TEM analyses have detected AgNPs in various parts of the root in *Festuca rubra*, including the cell wall and cortex of parenchymal cells [46]. Stomata in leaves of *Arabidopsis thaliana* have also been implicated in facilitating AgNP uptake and translocation [47], although it remains unclear whether AgNPs can move through the food chain [10,48]. In *Zea mays* (maize), TEM studies have demonstrated that CuNPs can be translocated from roots to shoots via the xylem, where they may aggregate or transform into other forms [49]. TEM imaging also revealed CuNPs (~25 nm) in soybean seeds, showing positive effects as they passed through the plasma membrane [50]. Additionally, CuNPs were observed to translocate from root to shoot in *Elsholtzia splendens* [51]. In summary, differences in the release rate and reactivity of Ag and Cu nanoparticles influence their uptake, translocation, and overall impact on plants [45,46,50].

### 3.3. Effect of Nanoparticles on the Phytochemical Composition of In Vitro O. petraeum

GC-MS analysis revealed that the volatile profiles of *O. petraeum* varied depending on nanoparticle treatment. Microshoots treated with 50 mg/L CuNPs (Table 3, Figure S1 (Supplementary Materials)) exhibited a profile dominated by α-Terpinyl Acetate (29.23%) and Geranyl Acetate (12.76%), along with Geraniol (7.18%), β-Caryophyllene (9.13%), and γ-Muurolene (7.16%), indicating enhanced geranyl derivative biosynthesis. Minor components such as Cyclopentadecanolide (3.81%) and Cedroxyde (1.74%) further emphasized the impact of CuNPs on terpenoid pathways. These findings align with Tabatabaee et al. (2021) [52], who reported that CuNPs can stimulate plant growth and secondary metabolism, while posing lower phytotoxicity than bulk copper. Similar effects of CuNPs on antioxidant activity and metabolite accumulation have been observed in *Ocimum basilicum* and *Bacopa monnieri* [18,53].

In contrast, treatment with 50 mg/L AgNPs (Table 4, Figure S2 (Supplementary File)) produced a sesquiterpene-rich profile, dominated by Caryophyllene Oxide (28.45%) and β-Caryophyllene (21.94%). Other notable compounds included α-Humulene (14.70%) and Humulene Epoxide II (11.40%), with minor constituents such as Geranial (2.94%) and Italicene Epoxide (2.93%). This indicates that AgNPs enhance sesquiterpene biosynthesis, consistent with previous findings in *Carum carvi* callus cultures, where AgNPs upregulated γ-terpinene synthase (CcTPS2) and increased secondary metabolites [54].

Under control conditions (30 g/L sucrose, no nanoparticles; Table 5, Figure S3 (Supplementary Materials)), microshoots showed a simpler profile, dominated by Caryophyllene Oxide (25.71%), n-Decanal (10.97%), and n-Nonanal (8.07%), with moderate amounts of α-Terpinyl Acetate (18.98%) and β-Caryophyllene (10.34%). This baseline volatile composition confirms that nanoparticles induce stress-related metabolic pathways, diversifying secondary metabolite production, as previously observed in *Mentha pulegium* and *Thymus* species [55,56].

The wild plant sample (Royal Botanic Garden, Jordan; Table 6, Figure S4 (Supplementary Materials)) displayed a profile dominated by monoterpenes and sesquiterpenes. Eudesm-7(11)-en-4-ol (25.10%) was the most abundant compound, followed by terpinen-4-ol (19.55%), β-Caryophyllene (12.20%), and abietatriene (7.13%), reflecting the plant’s natural chemotype. This has also been previously demonstrated in plants belonging to the Lamiaceae family, due to their content of the same bioactive compounds [57,58]. Environmental factors, such as stress and soil composition, likely contribute to this chemical diversity [59,60].

CuNPs and AgNPs can act as elicitors, specifically triggering stress responses in plants that enhance the production of secondary metabolites (e.g., flavonoids, phenolics, essential oils) [18,20,61]. In contrast, traditional chemical elicitors may be less selective or slower in inducing these pathways [40,62]. Additionally, nanoparticles can gradually release metal ions, providing a longer-lasting stimulus to the plant’s metabolism compared to the sudden burst from salts or other chemicals [63,64]. CuNPs and AgNPs can be taken up by plant tissues, especially in in vitro cultures [65]. However, studies show that most nanoparticles accumulate in roots and leaves, and their transfer into secondary metabolites (such as essential oils, flavonoids, or phenolics) is minimal or negligible [66]. Therefore, while nanoparticles stimulate metabolite production, they generally do not become part of the secondary metabolites [67]. This allows plants to respond at lower doses, reducing toxicity risks. Moreover, since lower doses are effective, using nanoparticles may result in less chemical runoff and lower environmental contamination compared to conventional metal salts or synthetic elicitors [68]. Proper extraction and purification further minimize any potential contamination, making health risks minimal when concentrations are controlled [66].

CuNPs and AgNPs were applied to culture media to evaluate their effects on microshoot growth and secondary metabolite production. Mechanistically, these nanoparticles act as elicitors, inducing mild oxidative stress that activates key enzymes involved in secondary metabolite biosynthesis, such as phenylalanine ammonia-lyase (PAL) [67,69]. Nanoparticles can enhance secondary metabolite production in in vitro cultures by inducing stress responses, modulating gene expression and enzyme activity, and acting as catalysts in biosynthetic pathways [40,70,71]. Their small size and high surface area allow efficient interactions with plant cells, enhancing nutrient uptake and influencing hormonal signaling pathways [69,72]. Recent studies [40,55,70,71,72] have reported similar effects of metal nanoparticles on plant tissue cultures, supporting the observed enhancement in metabolite production.

Collectively, these results provide a comprehensive understanding of how nanoparticles stimulate plant growth and secondary metabolism. They also highlight the potential of *O. petraeum* as a valuable source of bioactive compounds for pharmacological and biotechnological applications. Such effects underscore the promise of nanoparticles for enhancing the medicinal and industrial value of aromatic plants.

### 3.4. Effect of Nanoparticles on Nutrient Uptake in In Vitro O. petraeum Microshoots

As shown in Table 7, nanoparticle treatments significantly affected nutrient composition in *O. petraeum* microshoots. The control group (MS medium without nanoparticles) contained 3.15 mg/g P, 36.31 mg/g K, and 12.12 mg/g Ca, consistent with reported nutrient ranges in plant dry matter [73]. Microshoots treated with 50 mg/L CuNPs showed increased phosphorus (3.44 mg/g) but reduced potassium (24.98 mg/g) and calcium (3.75 mg/g). Similar effects of CuNPs on nutrient uptake have been reported in *Glycine max*, with phosphorus metabolism influenced by enzymatic regulation [37,50]. Conversely, 50 mg/L AgNPs increased calcium levels to 13.31 mg/g while decreasing phosphorus and potassium. AgNPs have been shown to enhance calcium uptake and other macronutrients in various plants, likely via stress-induced signaling, membrane stabilization, and increased nutrient bioavailability [11,74,75]. Overall, nanoparticle treatments influenced major element uptake, with CuNPs notably elevating phosphorus but reducing potassium and calcium, while AgNPs primarily increased calcium. These effects may result from enhanced root interaction, membrane permeability, and nutrient bioavailability induced by nanoparticles [72,76].

## 4. Conclusions

Nanoparticle treatments significantly enhanced growth, secondary metabolite production, and nutrient uptake in *O. petraeum* microshoots. CuNPs promoted phosphorus accumulation and geranyl derivatives, while AgNPs increased calcium levels and sesquiterpene biosynthesis. TEM analysis confirmed the absorption and movement of both nanoparticles within roots and leaves. This study is the first to report the influence of nanoparticles on the micropropagation of *O. petraeum* and its essential oil profile, highlighting the effect of culture conditions and nanoparticle type on chemical composition of plant metabolites. More studies are needed to improve nanoparticle use, enhance terpenoid yield, and assess long-term influence on plant development and genetic stability.

## Figures and Tables

**Table 1:** Influence of AgNPs on in vitro *O. petraeum* microshoot growth parameters after growing on MS medium enriched with various AgNPs concentrations and 0.5 mg/L GA_3_.

Ag NPs Concentration (mg/L)	Microshoot Number/Explant	Shoot Height (cm)	Root Numbers	Callus Diameter (cm)
0 ^x^	2.80 ± 0.37 c *	5.24 ± 0.12 c	2.60 ± 0.51 c	0.86 ± 0.50 d
25	3.80 ± 0.37 c	4.54 ± 0.05 d	3.80 ± 0.37 bc	2.00 ± 0.71 b
50	6.80 ± 0.37 b	5.94 ± 0.05 b	4.80 ± 0.37 b	1.54 ± 0.51 c
100	11.60 ± 0.50 a	9.22 ± 0.11 a	8.20 ± 0.40 a	1.84 ± 0.50 b
150	7.60 ± 0.51 b	6.18 ± 0.08 b	5.2 ± 0.37 b	2.38 ± 0.86 a

* Tukey’s HSD test shows that values with different letters within each column are significantly different at *p* ≤ 0.05. Data entries are presented as means ± standard error. ^x^ Control treatment.

**Table 2:** Influence of CuNPs on in vitro *O. petraeum* microshoot growth parameters after culturing on MS media enriched with various levels of CuNPs concentrations and 0.5 mg/L GA_3_.

Cu NPs Concentration (mg/L)	Microshoot Number/Explant	Shoot Height (cm)	Root Numbers	Callus Diameter (cm)
0 ^x^	2.80 ± 0.37 cd *	5.26 ± 0.06 b	3.20 ± 037 c	1.03 ± 0.37 c
25	4.80± 0.30 b	3.84 ± 0.09 c	5.60 ± 0.50 b	1.08 ± 0.58 c
50	6.80 ± 0.37 a	7.00 ± 0.31 a	13.80 ± 0.58 a	1.28 ± 0.37 c
100	3.20 ± 0.30 c	5.20 ± 0.37 b	6.00 ± 0.44 b	2.48 ± 0.86 a
150	1.60 ± 0.24 d	4.20 ± 0.30 bc	4.80 ± 0.58 bc	2.16 ± 0.92 b

* Tukey’s HSD test shows that values with different letters within each column are significantly different at *p* ≤ 0.05. Data entries are presented as means ± standard error. ^x^ Control treatment.

**Table 3:** Concentrations of chemical profile components in dried *O. petraeum* microshoots treated with copper nanoparticles (50 mg/L CuNPs).

SN *	RT **	RI_exp_^x^	RI_lit_^y^	Compound ^a^	%Content ^b^	Classification Group ^z^
1	9.831	1028	1024	Limonene	3.60	Monoterpene Hydrocarbon
2	15.100	1153	1155	iso-Isopulegol	4.94	Monoterpenoid Alcohol
3	19.057	1242	1235	Neral	4.64	Monoterpenoid Aldehyde
4	20.421	1273	1264	Geraniol	7.18	Monoterpenoid Alcohol
5	23.620	1346	1346	α-Terpinyl Acetate	29.23	Monoterpenoid Ester
6	24.930	1376	1379	Geranyl Acetate	12.76	Monoterpenoid Ester
7	26.517	1413	1417	β-Caryophyllene	9.13	Sesquiterpene Hydrocarbon
8	28.052	1450	1452	α-Humulene	4.91	Sesquiterpene Hydrocarbon
9	29.129	1476	1478	γ-Muurolene	7.16	Sesquiterpene Hydrocarbon
10	33.384	1581	1582	Caryophyllene oxide	1.73	Sesquiterpenoid Oxide
11	38.217	1709	1713	Cedroxyde	1.74	Sesquiterpenoid Oxide
12	42.543	1834	1832	Cyclopentadecanolide	3.81	Lactone
13	44.930	1908	1913	(5E,9E)-Farnesyl acetone	2.06	Sesquiterpenoid Ketone
14	51.161	2100	2087	Abietadiene	7.09	Diterpene Hydrocarbon

* SN: Compound serial number; ** RT: Retention time of the compound (minutes); ^x^ RI_exp: Linear retention index determined on a DB-5 equivalent column; ^y^ RI_lit: Reference retention index from literature (on a DB-5 equivalent column); ^a^ Compounds are presented according to their elution order; ^b^ Average of four measurements (two oil samples, each analyzed in duplicate) with standard deviation (SD) within ±5% of the mean. ^z^ Total Monoterpene Content: 62.35% (Monoterpene Hydrocarbons: 3.60%; Oxygenated Monoterpenes (have O group; (Alcohol; Aldehydes, Esters)): 58.75%). Total Sesquiterpene Content: 26.73% (Sesquiterpene Hydrocarbons: 21.20%; Oxygenated Sesquiterpenes: 5.53%). Miscellaneous Content: 10.90% (Lactone: 3.81%; Diterpene Hydrocarbon: 7.09%).

**Table 4:** Concentrations of chemical profile components in *O. petraeum* microshoots treated with silver nanoparticles (50 mg/L Ag NPs).

SN *	RT **	RI_exp_^x^	RI_lit_^y^	Compound ^a^	%Content ^b^	Classification Group ^z^
1	6.475	930	932	α-Pinene	2.58	Monoterpene Hydrocarbon
2	7.901	975	974	β-Pinene	1.83	Monoterpene Hydrocarbon
3	19.063	1242	1235	Neral (z- citral)	1.98	Monoterpenoid Aldehyde
4	20.413	1273	1265	Geranial (E-citral)	2.94	Monoterpenoid Aldehyde
5	20.746	1280	1283	Isobornyl acetat	1.00	Monoterpenoid Ester
6	23.574	1345	1346	α-Terpinyl Acetat	1.46	Monoterpenoid Ester
7	26.516	1413	1417	β-Caryophyllene	21.94	Sesquiterpene Hydrocarbon
8	28.057	1450	1452	α-Humulene	14.70	Sesquiterpene Hydrocarbon
9	29.125	1476	1478	g-Muurolene	4.76	Sesquiterpene Hydrocarbon
10	29.760	1491	1500	bicyclogermacrene	1.46	Sesquiterpene Hydrocarbon
11	32.060	1548	1547	Italicene epoxide	2.93	Sesquiterpenoid Oxide
12	33.369	1580	1582	Caryophyllene oxide	28.45	Sesquiterpenoid Oxide
13	34.528	1610	1608	Humulene epoxide II	11.40	Sesquiterpenoid Oxide
14	42.596	1836	1832	Cyclopentadecanone	2.57	Lactone

* SN: Compound serial number; ** RT: Retention time of the compound (minutes); ^x^ RI_exp: Linear retention index determined on a DB-5 equivalent column; ^y^ RI_lit: Reference retention index from literature (on a DB-5 equivalent column); ^a^ Compounds are arranged according to their elution sequence; ^b^ Mean values from four measurements (two oil samples, each analyzed in duplicate) with standard deviation (SD) within ±5% of the mean. ^z^ Total Monoterpene Content: 11.79% (Monoterpene Hydrocarbons: 4.41%; Oxygenated Monoterpenes: 7.38%) Total Sesquiterpene Content: 85.64% (Sesquiterpene Hydrocarbons: 42.86%; Oxygenated Sesquiterpenes: 42.78%) Miscellaneous Content: 2.57% (Lactone 2.57%).

**Table 5:** Concentrations of chemical profile components in dried *O. petraeum* microshoots cultured on MS medium containing 0.5 mg/L GA_3_ and 30 g/L sucrose (Control 1).

SN *	RT **	RI_exp_ ^x^	RI_lit_ ^y^	Compound ^a^	%Content ^b^	Classification Group ^z^
1	12.940	1103	1100	n-Nonanal	8.07	Aldehyde
2	17.403	1205	1201	n-Decanal	10.97	Aldehyde
3	23.534	1344	1346	α-Terpinyl Acetat	18.98	Monoterpenoid Ester
4	26.483	1412	1417	β-Caryophyllene	10.34	Sesquiterpene Hydrocarbon
5	28.035	1449	1452	α-Humulene	9.54	Sesquiterpene Hydrocarbon
6	33.316	1579	1582	Caryophyllene oxide	25.71	Sesquiterpenoid Oxide
7	38.194	1708	1713	Cedroxyde	16.39	Sesquiterpenoid Oxide

* SN: Compound serial number; ** RT: Compound retention time (minutes); ^x^ RI_exp: Linear arithmetic retention index measured on a DB-5 equivalent column; ^y^ RI_lit: Reference arithmetic retention index from literature (measured on a DB-5 equivalent column); ^a^ Compounds are listed according to their elution order; ^b^ Mean of four determinations (two oil samples, each analyzed in duplicate) with standard deviation (SD) within ±5% of the mean.^z^ Total Monoterpenoid Content: 18.98% (Oxygenated Monoterpenes: 18.98%; Hydrocarbon Monoterpenes: 0.00%). Total Sesquiterpene Content: 61.98% (Sesquiterpene Hydrocarbons: 19.88%; Oxygenated Sesquiterpenes: 42.10%). Miscellaneous Content: 19.04% (Aldehydes: 19.04%).

**Table 6:** Concentrations of chemical profile components in dried wild *O. petraeum* aerial (Control 2).

SN *	RT **	RI_exp_ ^x^	RI_lit_ ^y^	Compound ^a^	%Content ^b^	Classification Group ^z^
1	9.265	1014	1014	α-Terpinene	2.56	Monoterpene Hydrocarbon
2	10.956	1055	1054	γ-Terpinene	4.70	Monoterpene Hydrocarbon
3	16.801	1191	1174	Terpinen-4-ol (mix 1174)	19.55	Monoterpenoid Alcohol
4	21.308	1293	1299	Terpinen-4-ol acetate	4.22	Monoterpenoid Ester
5	26.527	1413	1417	β-Caryophyllene	12.20	Sesquiterpene Hydrocarbon
6	29.088	1475	1478	γ-Muurolene	4.61	Sesquiterpene Hydrocarbon
7	30.163	1500	1505	β-Bisabolene	3.42	Sesquiterpene Hydrocarbon
8	30.751	1515	1522	δ-Cadinene	4.75	Sesquiterpene Hydrocarbon
9	33.351	1580	1582	Caryophyllene oxide	7.16	Sesquiterpenoid Oxide
10	37.827	1698	1700	Eudesm-7(11)-en-4-ol	25.10	Sesquiterpenoid Alcohol
11	42.560	1834	NA ^m^	6,10,14-trimethyl-2- Pentadecanone (Or Unk HC)	2.53	Hydrocarbon
12	43.326	1859	1867	(E)-β-Santalol acetate	2.07	Sesquiterpenoid Ester
13	49.669	2053	2055	Abietatriene	7.13	Diterpene Hydrocarbon

* SN: Compound serial number; ** RT: Compound retention time (minutes); ^x^ RI_exp: Linear arithmetic retention index measured on a DB-5 equivalent column; ^y^ RI_lit: Reference arithmetic retention index from literature (measured on a DB-5 equivalent column); m: Not identified; ^a^ Compounds are listed according to their elution order; ^b^ Mean of four determinations (two oil samples, each analyzed in duplicate) with standard deviation (SD) within ±5% of the mean. ^z^ Total Monoterpene Content: 31.03% (Monoterpene Hydrocarbons: 7.26%; Oxygenated Monoterpenes: 23.77%). Total Sesquiterpene Content: 59.31% (Sesquiterpene Hydrocarbons: 25.98%; Oxygenated Sesquiterpenes: 34.33%). Miscellaneous Content: 9.66% (Hydrocarbon: 2.53%; Diterpene Hydrocarbon: 7.13%).

**Table 7:** Effect of Nanoparticles on Nutrients Uptake in *Origanum petraeum* Microshoots.

Sample		Element (mg/g)	
	P	K	Ca
Control ^x^	3.15133 ± 0.00491 b *	36.31067± 0.00348 a	12.11567 ± 0.00348 b
50 mg/L CuNPs	3.43533 ± 0.00819 a	24.97567± 0.00348 b	3.75133 ± 0.0041 c
50 mg/L AgNPs	2.95233 ± 0.01178 c	24.41100± 0.00665 c	13.310 ± 0.00577 a

* Tukey’s HSD test shows that values with different letters within each column are significantly different at *p* ≤ 0.05. Data entries are presented as means ± standard error. ^x^ Control treatment.

## Data Availability

The data produced and examined in this study can be obtained from the corresponding authors upon request for research purposes.

## Supplementary Materials

The following supporting information can be downloaded at: https://www.mdpi.com/article/10.3390/nano15191496/s1. Figure S1: GC-MS chromatogram showing the volatile components in *O. petraeum* microshoots treated with copper nanoparticles (50 mg/L Cu NPs).; Figure S2: GC-MS chromatogram showing the volatile components in *O. petraeum* microshoots treated with silver nanoparticles (50 mg/L Ag NPs).; Figure S3: GC-MS chromatogram showing the volatile components in *O. petraeum* microshoots on MS medium with GA_3_ at 0.5 mg/L and sucrose at 30 g/L (Control 1).; Figure S4: GC-MS chromatogram showing the volatile components in wild *O. petraeum* aerial parts (Control 2).
