# Supplementary material for: Nanoparticles Enhance In Vitro Micropropagation and Secondary Metabolite Accumulation in Origanum petraeum
Source: Nanomaterials (Basel). 2025 Sep 30;15(19):1496. doi: 10.3390/nano15191496 (PMC12526291; doi:10.3390/nano15191496)
Supplement: Supplementary file 1 [file nanomaterials-15-01496-s001.zip › nanomaterials-3893715-supplementary.pdf]

Supplementary Materials

Nanoparticles Enhance in Vitro Micropropagation and Secondary Metabolite Accumulation in*Origanum petraeum*

Tamara S. Al Qudah [1], Rida A. Shibli [1,2], Rund Abu-Zurayk [1,*] and Mohammad Hudaib [3]

[1] Hamdi Mango Center for Scientific Research, The University of Jordan, Amman P.O. Box 11942, Jordan
[2] Department of Horticulture and Crop Sciences, Faculty of Agriculture, University of Jordan, Amman P.O. Box 11942, Jordan
[3] Faculty of Pharmacy, The University of Jordan, Amman 11942, Jordan
* Correspondence: r.abuzurayk@ju.edu.jo

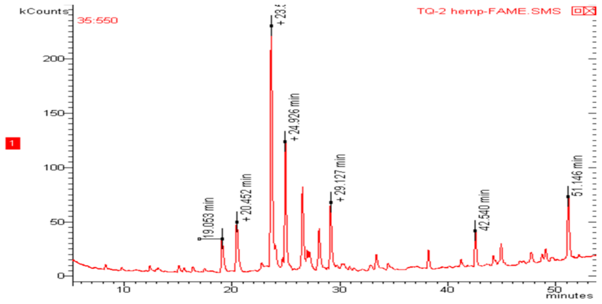

Figure S1. GC-MS chromatogram showing the volatile components in *O. petraeum* microshoots treated with copper nanoparticles (50 mg/L Cu NPs).

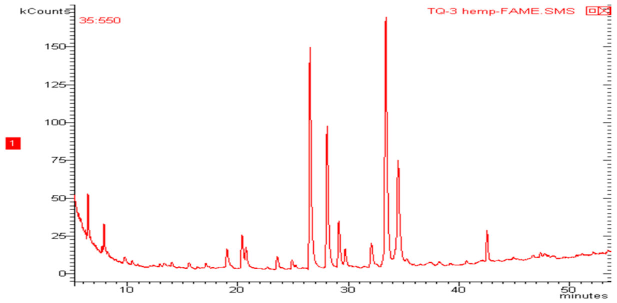

Figure S2. GC-MS chromatogram showing the volatile components in *O. petraeum* microshoots treated with silver nanoparticles (50 mg/L Ag NPs).

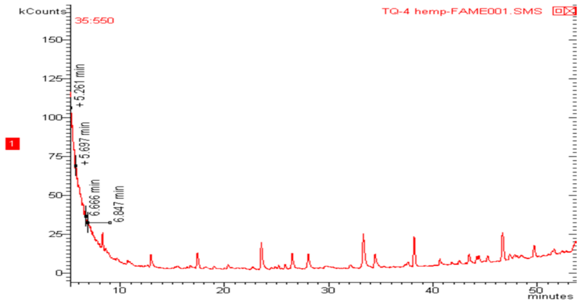

Figure S3. GC-MS chromatogram showing the volatile components in *O. petraeum* microshoots on MS medium with GA_3 at 0.5 mg/L and sucrose at 30 g/L (Control 1).

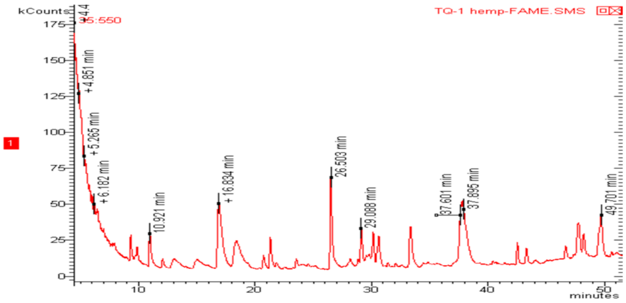

Figure S4. GC-MS chromatogram showing the volatile components in wild *O. petraeum* aerial parts (Control 2).
